# Dermoid Cyst of the Penis: A Case Report of an Unusual Penile Mass

**DOI:** 10.7759/cureus.30227

**Published:** 2022-10-12

**Authors:** Mallipeddi Partha Sri, Arvind Kumar Prabhat, Mahesh Rahul Dammalapati, Kapang Yirang, Bala Murali Krishna Patibandla, Ketan Kantamaneni, Lakshmi Malvika Atluri, Ziad El Menawy, Surya Kumari Uppalapati

**Affiliations:** 1 Urology, Dr. Pinnamaneni Siddhartha Institute of Medical Sciences and Research Foundation, Gannavaram, IND; 2 Surgery, Dr. Pinnamaneni Siddhartha Institute of Medical Sciences and Research Foundation, Gannavaram, IND; 3 Internal Medicine, Zayed Hospital, Abu Dhabi, ARE

**Keywords:** median raphe cysts, cystic hamartoma, surgery general, urology surgery, dermoid cysts, penile cyst, penile mass

## Abstract

Dermoid cysts are subcutaneous swellings that are usually congenital and originate from the sequestration of embryonic epithelium along the lines of embryonic closure. They are composed of a mixture of sebaceous fluid, keratin, cholesterol crystals, calcium, hair follicles, sweat glands, and sebaceous glands. They present as a non-tender mass that is well-circumscribed, firm in consistency, and usually asymptomatic. Occasionally, dermoid cysts can get infected and form an abscess. Surgical excision remains the linchpin of treatment for dermoid cysts. Dermoid cysts are most common on the head, face, neck, and thoracoabdominal region and are very rare on the prepuce (foreskin). We report the case of a 27-year-old male who presented with a midline penile mass, difficulty in retracting his prepuce, and painful intercourse. A basic hematological and radiological workup was done to rule out the other differentials. Surgical excision of the swelling was done, and a histopathology report proved it to be dermoid. This case report highlights the possibility of the presence of a dermoid at rare anatomical locations such as the penis.

## Introduction

Penile swellings are a relatively rare phenomenon, and they can be present on the shaft of the penis, glans penis, and the prepuce [1]. Dermoid cysts are subcutaneous swellings, which are usually congenital and originate as a result of the sequestration of embryonic epithelium along the lines of embryonic closure [2]. They are most commonly present at the lateral one-third of the eyebrow. They are composed of a mixture of sebaceous fluid, keratin, cholesterol crystals, calcium, hair follicles, sweat glands, and sebaceous glands. They derive from mesoderm (adnexal structures) and ectoderm (squamous epithelium). Dermoid cysts are rarely seen on the external genitalia, and a meager number of cases were noted.

They present as a non-tender mass that is well-circumscribed, firm in consistency, and usually asymptomatic [3]. Dermoid cysts tend to be dormant for a time and may later continue to grow or even extend into surrounding structures. Our patient is a 27-year-old male who presented to the urology department with a chief complaint of an asymptomatic swelling over his penis since childhood which was diagnosed as a dermoid cyst. A handful of reports in the literature have described a dermoid cyst in the penis as its occurrence is infrequent considering its anatomical location. The purpose of the case report is to raise awareness among surgeons regarding various presentations of penile masses and to raise the suspicion of a dermoid cyst as a differential diagnosis with midline penile masses.

## Case presentation

History and examination

A 27-year-old gentleman presented to the urology outpatient department at our hospital with swelling over his penis since childhood. Additionally, the patient had difficulty retracting the prepuce and had painful intercourse. The swelling gradually increased and obtained its current size. There were no periods of any rapid increase in its size. The swelling was not associated with pain, discharge, hematuria, dysuria, and inflammation. There was no history of any infection or trauma in the past. His urinary flow and stream were good, and there was no history of discharge per urethra and fever. The patient did not have any significant past medical history or personal history. There were no similar complaints from any family member.

**Figure 1:**
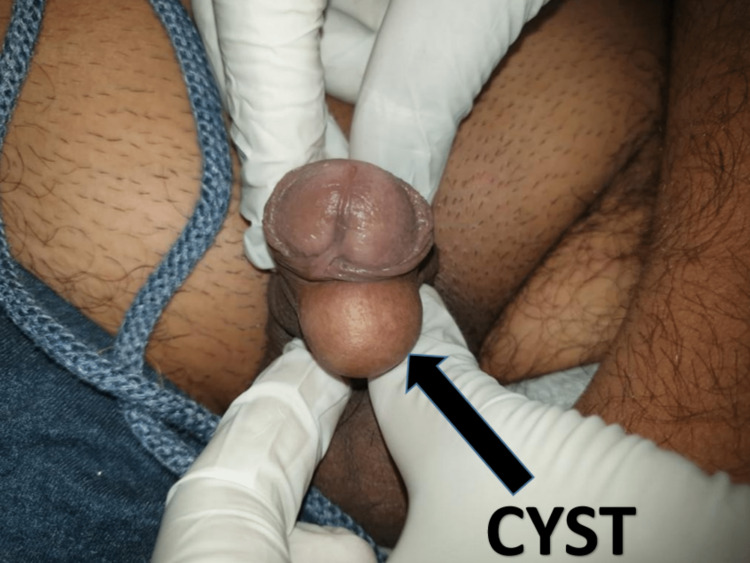
Swelling in the ventral aspect of the penis

On further examination, a solitary 4 x 4 cm, spherical-shaped swelling was identified on the ventral aspect of the preputial skin anterior to the coronal sulcus (Figure 1).

The swelling was fluctuant, non-tender, non-compressible, and non-pulsatile, with the skin over the swelling being smooth and cystic in consistency. The transillumination test and cough impulse were negative. Inflammatory signs such as tenderness, warmth, and redness were absent. There was no associated lymphadenopathy.

Investigations

A complete basic surgical profile workup was initially done, including a complete blood count and a complete metabolic panel where all the values were within the normal range (Table 1).

**Table 1:** Laboratory workup

Laboratory investigation	Laboratory value	Reference range
WBC Count	9.70	4.50-11.00 X 10*3/uL
Haemoglobin	14.0	12.0-15.7 g/dL
Platelet count	220	140-440 X 10*3/uL
ABS Neutrophils	4.82	1.40-6.50 X 10*3/uL
Creatinine	0.90	0.50-1.50 mg/dL
Blood Urea Nitrogen	16	9-27 mg/dL
Albumin	4.0	3.8-4.9 d/dl
C Reactive protein	0.2	0.0-0.9 mg/dL

**Figure 2:**
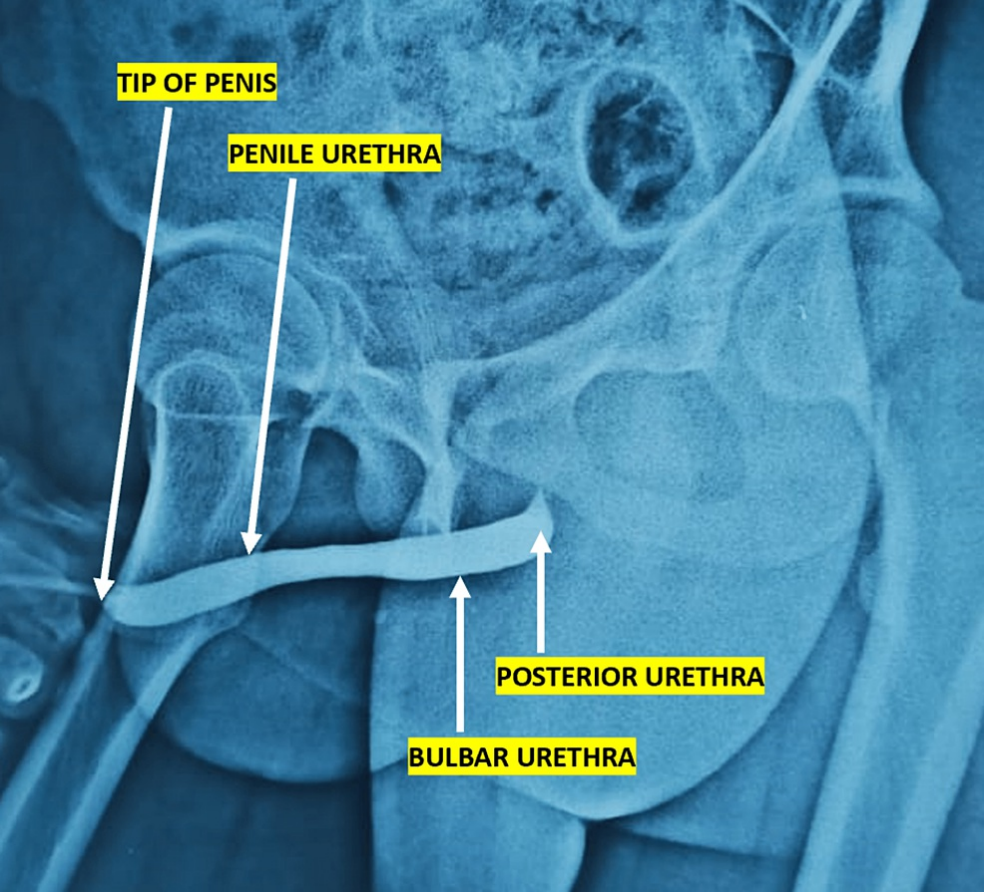
Normal retrograde urethrogram

Viral markers, urine dipstick, microscopy, and culture were negative. A retrograde urethrogram was done to rule out the urethral diverticulum (Figure 2).

Differential diagnosis

The possible differential diagnoses of penile skin lesions are glomus tumours, pilonidal cysts, epidermal inclusion cysts, median raphe cysts, urethral diverticula, and steatocystomas originating in the genital region. 

Treatment and prognosis

**Figure 3:**
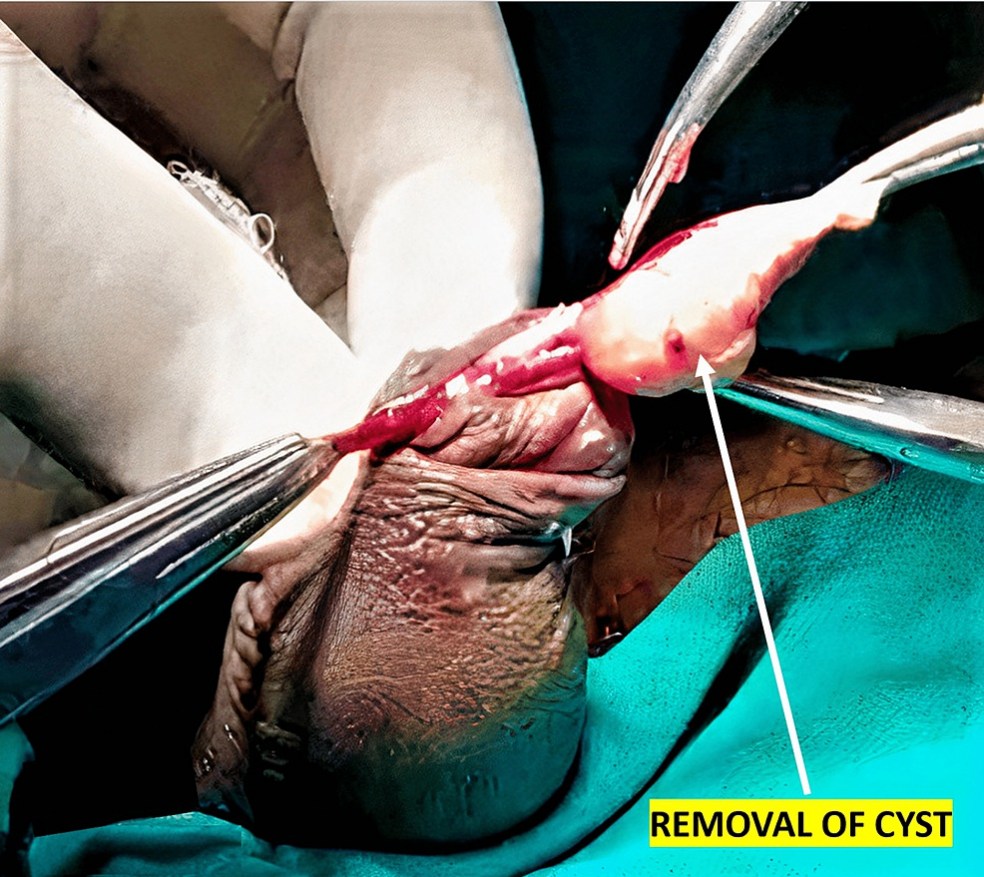
Intra-operative picture showing cyst being removed

The mainstay of treatment is the surgical excision of the cyst (Figure 3).

**Figure 4:**
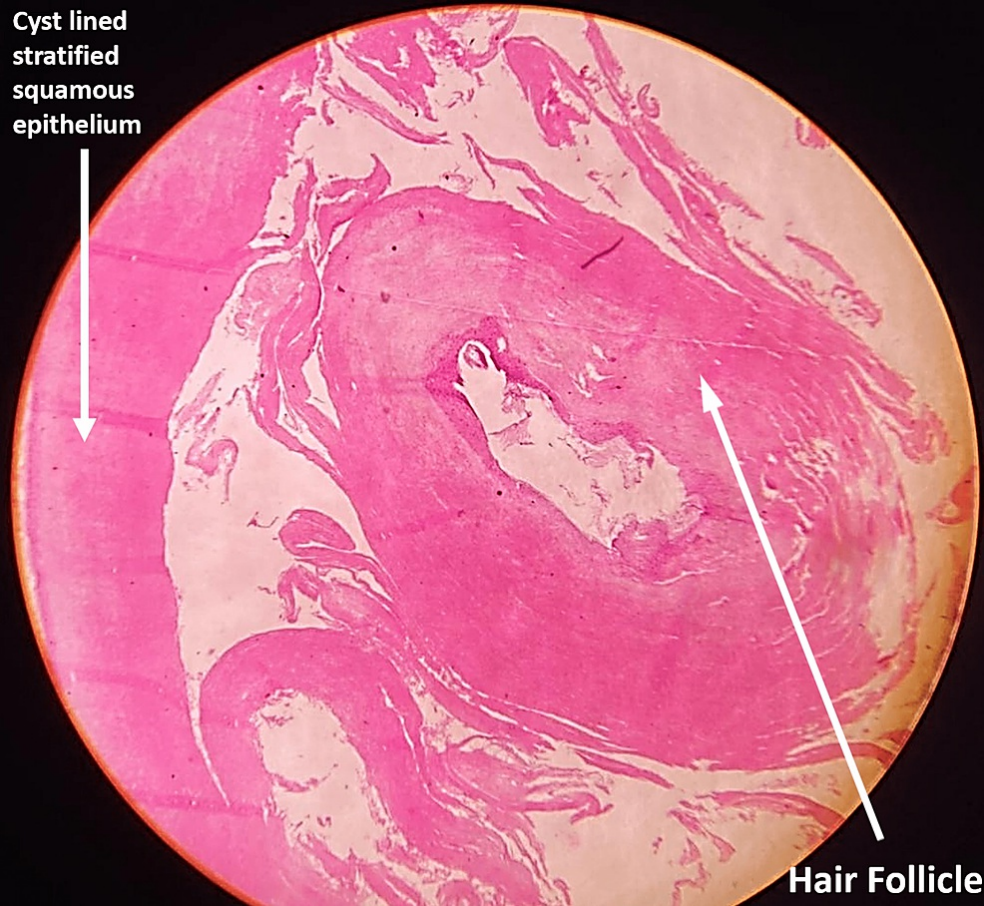
Histology showing cyst lined by squamous epithelium, filled with keratinous material and cross sections of hair shafts, suggestive of dermoid cyst

To confirm the diagnosis of a dermoid cyst, the specimen was sent for histopathological examination (Figure 4).

It was revealed that the cyst was lined by squamous epithelium and comprised of keratinous material and cross-sections of hair shafts suggestive of a dermoid cyst.

No recurrence was seen during a follow-up period of two years. Dermoid cysts have a good prognosis overall.

## Discussion

Swellings over the penis are an unconventional entity. Other plausible cutaneous lesions are sebaceous cysts, urethral diverticulum or a urethral calculus, dermoid cysts, or malignancies like squamous cell carcinoma, secondary deposits, and sarcoma. They rarely can also be a plaque of the tunica [4]. Dermoid cysts can be considered to be true hamartomas. They occur due to skin and its structures getting trapped during the development of the fetus [2,3]. After getting trapped in the subcutaneous plane, the dermal cells continue proliferating and liquefy to form a cyst. The cleft hair can get trapped at the coronal sulcus and form a dermoid cyst. A natural movement drives this hair into the shaft or prepuce [2]. Dermoid cysts are most common on the head, face, neck, and thoracoabdominal region and are very rare on prepuce [1]. Dermoid cysts are germ cell derivatives and contain skin and appendages [4]. The dermoid cyst can be differentiated from an epidermoid cyst by the presence of dermal appendages, stratified squamous epithelium lining, and surrounded by penile tissue and not merely keratin upon histopathological examination [5].

Dermoid cysts are usually cutaneous or subcutaneous nodules, which can occasionally get infected and form an abscess [2]. Surgical excision remains the central component of treatment [1,3,6]. Small penile swellings are asymptomatic. More prominent ones result in morbidity because of the inability of preputial retraction, painful intercourse, secondary infections, urethral obstruction, and cosmetic concerns, which warrant a surgical intervention [1,5,6].

A median raphe cyst of the penis can be significant when considering other differential diagnoses. They usually occur on the ventral aspect of the glans penis. Their occurrence is also rare, like the dermoid cyst, and is generally asymptomatic. They can be present since childhood and grow slowly like a dermoid cyst. A dermoid can be differentiated from a median raphe cyst primarily by the site and upon histopathological examination. Median raphe cysts are commonly present in the midline in the ventral aspect of male external genitalia ranging from the external urethral meatus to the anus. They are lined by stratified layers of columnar to cuboidal epithelial cells [7].

There are no specific anatomical location boundaries for dermoid; they can also occur in the scrotum and perineum. Dermoid can be a rare cause of an intrascrotal mass, and they arise extratesticularly, and extend deep into the pelvis infrequently. A few case reports describe that dermoid can arise from the testes themselves. When they occur as an intrascrotal mass, the factors that facilitate their diagnosis are a non-vascularised appearance on ultrasound, and the presence of sebaceous material during surgical exploration [8].

## Conclusions

Dermoid cysts are rare on the external genitalia, and only a few cases are reported. Generally, they present as cystic swellings in the subcutaneous region due to the sequestration of cutaneous tissues along the embryonal lines of closure at the lateral third of the eyebrows, nose, and scalp. They mainly comprise keratin and sebaceous fluid. This case report aims to highlight the possibility and ignite the notion of a dermoid cyst when a midline mass on the shaft of the penis or glans penis is present. Clinically, they can be distinguished by the presence of a well-circumscribed, firm, non-tender, and non-erythematous mass. The cornerstone of the diagnosis lies in the gross examination and histopathological examination of the resected specimen. When small in size, they tend to be asymptomatic but can occasionally get infected and pose problems during micturition and intercourse when large. The most common differential diagnosis, such as median raphe cysts, pilonidal cysts, epidermal inclusion cysts, and urethral diverticula, should be ruled out. A retrograde urethrogram should be done to rule out a urethral diverticulum. Surgical excision of the cyst remains the chief modality of treatment.
